# In Situ, Real-Time Temperature Mapping and Thermal FE Simulations of Large-Format 3D Printed PETG/CF Vertical Wall

**DOI:** 10.3390/ma16196486

**Published:** 2023-09-29

**Authors:** Felipe Robles Poblete, Matthew Ireland, Lucinda Slattery, William G. Davids, Roberto A. Lopez-Anido

**Affiliations:** 1 Advanced Structures and Composites Center (ASCC), University of Maine, Orono, ME 04469, USA; felipe.robles@maine.edu (F.R.P.); matthew.ireland@maine.edu (M.I.); 2 Department of Physics and Astronomy, University of Maine, Orono, ME 04469, USA; lucinda.slattery@maine.edu; 3 Department of Civil and Environmental Engineering, University of Maine, Orono, ME 04469, USA; william.davids@maine.edu

**Keywords:** additive manufacturing, finite element, modeling, conductance, convection, additive manufacturing, thermoplastic, polymer

## Abstract

This work focuses on simulating the thermal history of a vertical wall consisting of a thermoplastic composite material, poly(ethylene terephthalate) glycol (PETG) with short carbon fiber reinforcement, manufactured using a Big Area Additive Manufacturing (BAAM) system. The incremental deposition process used in additive manufacturing, which corresponds to the repeated deposition of hot material onto cooler material, contributes to the presence of residual stresses and part warping. The prediction of these mechanisms is dependent on thermal history of the part, and the major motivation of this work was to improve the accuracy of finite element (FE) models used to quantify the thermal history of large-format additively manufactured parts. Thermocouples were placed throughout the part at varying heights to measure temperature as a function of time. The FE model developed found a thermal contact conductance between the printed part and the bed of 10 W/m^2^K and convection coefficient values that linearly varied from 3 to 15 W/m^2^K through the wall height when making a temperature comparison with the output from the thermocouples. It is also demonstrated that the FE model with a constant convection coefficient under-predicts model temperature at the beginning of the manufacturing process when compared against the model with a variable convection coefficient. The impact of this difference was seen in the stress values, which were larger for the model with a constant convection coefficient. Finally, a correlation equation was derived which allows the findings to be generalized to other vertical structures manufactured on the BAAM. In summary, this work offers valuable insights on material characterization, real-time thermocouple placement, and FE modeling of large-format additively manufactured parts.

## 1. Introduction

Large-format extrusion-based additive manufacturing is a technology that, in recent years, has become widespread in the fabrication of composite applications in the marine and construction industries, allowing for greater design flexibility while reducing lead times and costs [1,2,3]. The Big Area Additive Manufacturing (BAAM) [4] system developed at Oak Ridge National Laboratory in collaboration with Cincinnati Inc. has been successfully used to manufacture large parts with a variety of thermoplastic composites [5,6]. Both the BAAM system and desktop-scale Fused Filament Fabrication (FFF) extrude heated thermoplastic material along programmed tool paths to manufacture parts on a layer-by-layer basis [6]. Unlike FFF, BAAM uses a single-screw extruder to melt polymer pellets and force the molten material through a nozzle via a pressure differential [7] instead of resistively melting a thin filament feedstock. Single-screw extruders enable the use of thermoplastic materials at a relatively lower cost and at faster deposition rates with mass throughputs up to 50 kg/h. With regard to the architecture of manufactured parts, BAAM- and FFF-produced components are similar, although the former produces parts which are an order of magnitude larger with bead dimensions at or above 10 mm. An example of a large-format additively manufactured structure is BioHome3D, which is a 56 m^2^ modular house manufactured using a recyclable biopolymer filled with wood fiber [8].

Performance objectives for 3D-printed parts create demand for materials to exhibit functionalities [9] including improved electrical and thermal conductivity, mechanical strength, and stiffness at relatively low cost [5,10]. To accommodate this demand, researchers have attempted to mix different types of fillers, such as metal [10], glass fibers [11], and vapor grown short carbon fibers [11,12] into the polymer matrix. Although the macrostructure of large 3D-printed parts alone contributes to thermal and mechanical anisotropy due to layer-wise deposition [12,13], this phenomenon is accentuated in short-fiber composites. Fibers with varying aspect ratios tend to align in the print direction, significantly affecting homogenized material properties [7]. Moreover, fiber alignment has also been seen to vary within the printed bead itself [14].

For a fiber-reinforced thermoplastic polymer, the cooling behavior of the deposited material is governed by heat transfer to the environment due to convective and radiative heat losses as well as conduction between beads and layers [15]. The rate of cooling governs both the phase change from viscoelastic fluid to solid and inter-bead bond quality [16]. The combination of these processes impacts the formation of residual stresses and deformations within the part [17], affecting the shape of the extrudate [18] and subsequent mechanical properties [19,20]. High-quality characterization of the associated thermal history is therefore required to ensure robust prediction of outcomes from the manufacturing process. Moreover, new additive manufacturing technologies such as 4D printing rely on the programming of different extrudate temperatures throughout printing to govern the polymer structure shape transformation over time [21,22].

Experimentally obtained thermal history data are often captured and reported by means of infrared (IR) radiation thermography [14,23,24,25]. The initialization of an IR camera for data capture, which is a necessary component of thermographic measurement for accurate temperature reporting, requires information about the scene and subject to correctly correlate as-measured radiance with as-reported temperatures. This requisite information includes an emissivity parameter, which varies according to the material surface roughness, the temperature dependence of the material’s emissive response, the angle of incidence between the subject surface normal and camera optical axis, and the line-of-sight distance between subject and camera. Similarly, knowledge of the scene temperature is required during initialization to accurately account for the proportion of as-measured radiance due to reflection from the subject [26]. These factors are often neglected or simplified, as in the case of a constant emissivity value, the practice of which imposes error on the temperature data reported by IR cameras [25]. By contrast, thermocouples are commonly used in research and industry to measure temperature with relatively simple sources of inaccuracy and across different processes. Previously, thermocouples have been embedded in small-scale additively manufactured parts for in situ temperature characterization [27,28].

Predictive tools that incorporate the coupled impacts of bed temperature, ambient temperature, and material properties on thermal history are also necessary. Layer-by-layer deposition models have been developed ranging from simple axisymmetric 1D transient heat transfer models [14,29] to 3D finite element models (FE) [30,31]. The finite difference method has also been used to numerically model temperature variation for the FFF processes, including large-format additive manufacturing, due to reduced computation costs when compared against FE implementations [32,33,34]. Recently, a coupled thermo-mechanical numerical model to determine a suitable combination of the parameters that avoids the collapse of the deposited layer under self-weight was developed [35]. FE-based methods have represented the thermal history of 3D-printed parts based on element activation [36], accounting for temperature-dependent material properties [37] and presented features that allow modeling heat transfer at time scales small enough to capture rapid cooling events [38]. In particular, the commercial FE software Abaqus (https://www.3ds.com/products-services/simulia/products/abaqus/, accessed on 1 August 2023) with additive manufacturing capabilities has been used to model complex 3D-printed parts, such as cellular structures with homogenized material properties [39] and thin-walled tubular structures [40]. Abaqus has also been applied to model the thermal history, final deformed shape, and residual stresses in additively manufactured parts comprised of acrylonitrile butadiene styrene (ABS) polymer [41], ABS with short carbon fibers [15,42], polyphenylene sulfide (PPS) polymer with carbon fibers [43,44], and metals [45]. The majority of published research on the topic of thermal modeling for FFF rely on the use of a constant convection coefficient.

This work combines in situ temperature measurements obtained from an additively manufactured part with candidate FE models of the manufacturing process. Candidate models were compared against experimental data, and the FE implementation that minimized error was found to require a non-constant convection coefficient in order to accurately capture the thermal history of the part. Finite element analysis was used to model the complete thermal history of a large-format 3D printed vertical wall made of poly(ethylene terephthalate) glycol (PETG) with short carbon fiber (CF) reinforcement. PETG is recognized for its manufacturability with glass transition and melting temperatures of 85 °C and 260 °C, respectively [46,47], qualifying the material as a good candidate for thermal and structural characterization. The accuracy of the thermal model was enhanced by real-time temperature data gathered by thermocouples embedded in the part during the manufacturing process. The temperature correlation between experimentally obtained and numerically generated data facilitated the characterization of conductance between the part and print bed, as well as convective heat transfer between the part and the environment, comprising process model features which were found to substantially impact the development of residual stresses. Finally, a correlation equation was derived based on the analysis of the wall manufactured with PETG/CF material and tested on a separate wall manufactured with ABS/CF. The necessity of this study is driven by the tendency for large-scale additively manufactured parts to fracture and/or develop significant distortion during manufacturing due to the accumulation of residual stresses [48,49,50,51]. Hence, the objective of this work is to improve the accuracy of FE models intended to capture the thermal behavior of large-scale polymer AM during fabrication via in situ temperature measurements.

## 2. Materials and Methods

### 2.1. Printing Process Information

Part manufacturing was executed on the BAAM machine stationed in the Advanced Structures and Composites Center at the University of Maine campus in Orono, Maine. A prismatic vertical wall was chosen for geometric simplicity and to facilitate parametric convection studies via the measurement of temperature variations along the height. The wall was manufactured with Techmer Electrafil 1711 PETG, which is compounded with 18% carbon fiber volume fraction. The magnitude of fiber volume fraction was not chosen to satisfy any specific criteria, but it is typical for materials provided by the supplier, and prior studies have used products with similar amounts [14,25]. The average carbon fiber length and diameter were 163 µm and 7 µm, respectively. The wall consisted of a single bead with the first layer extended laterally to form a brim for improved stability. The initial manufacturing process parameters were based on the layer time utilized in a prior publication with similar geometry [14] and modified to mitigate the overall deformation and debonding between layers. The wall dimensions and manufacturing process parameters are given in Table 1.

Type K thermocouples were manually installed between layers of the wall to capture the temperature at the interfaces. Thermal history was obtained below the first layer at the part/bed interface and at layers 38, 77, 116, 155, and 194. A plywood scaffolding structure was designed to hold the thermocouple leads and utilized to prevent forces due to gravity from pulling thermocouples out of position during solidification of the extrudate.

### 2.2. Interlayer Thermocouples—Final Position Measurement

Although the approximate locations of embedded thermocouples were known from visual inspection, accurate positional measurements were taken to verify the quality of contact with the extrudate. A Quantum Max FaroArm^®^ (Faro Technologies, Lake Mary, FL, USA) was used to provide a detailed 3D scan of each wall for X–Z (length, height) location determination. A coordinate system was chosen to denote the locations of the thermocouples, and the planar geometry of the walls was leveraged accordingly. The coordinate system origin in X, Y and Z was chosen to be the first identifiable point where extrusion begins, the midpoint of the wall thickness, and the center point at the part/bed interface, respectively. Thermocouple locations are reported in reference to this coordinate system and were used for comparison with model data.

End-mill removal of the as-manufactured material revealed bond quality with the surrounding polymer as well as the relative location of the thermocouple within the bead. Overall, 30% of the thermocouples embedded in the PETG/CF wall exhibited some aspect of poor-quality bonds (C3, AI0, and AI3) as determined by visual inspection of the contact between the thermocouple lead wires and the extrudate. The set of thermocouples observed to have good bonds with their surrounding polymer was used as sources for comparison with model data.

**Figure 1:**
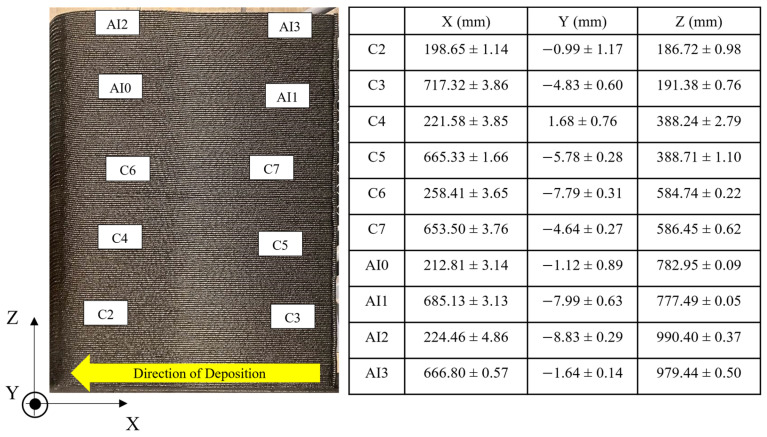
Measured positions of each interlayer thermocouples on the PETG/CF wall.

Figure 1 shows the position coordinates together with their calculated uncertainty values and labels for each interlayer thermocouple in the PETG/CF wall with respect to the direction that material was deposited in a given layer. Location uncertainty was characterized by disruptions in the external surface of the extrudate, which created regions of scan data devoid of information due to occlusion. Positions were determined by averaging the extreme values of the disruption in X, Y, and Z orientations. Finally, the average was then subtracted from the maximum value to determine the associated uncertainty. Thermocouples labeled as C0 and C1 were placed at the part/bed interface and are not included in Figure 1. The letter difference in thermocouple labels (C and AI) denotes sampling rates of 1 and 2 Hz, respectively.

### 2.3. PETG/CF Material Characterization

The material characterization procedures utilized in this work were based on a proposed roadmap for testing the same type of additively manufactured short-fiber composite materials [52]. Thermomechanical and mechanical property data were obtained as inputs to the FE models. Material property data were generated from test specimens exercised from a different part manufactured in PETG/CF with the same deposition temperature profile, deposition speed, and nominal bead dimensions as the wall print. Aligning the processing conditions in parts manufactured for material property characterization with the processing conditions employed for experimental prints is intended to control for uncharacterized process effects.

Density measurements were performed with the Specific Gravity Method according to the ASTM D792-20 [53]. In total, there were 54 samples (18 samples cut in each X, Y, and Z orientation). Each sample was 17 × 17 × 7 mm^3^ with the short axis parallel to the orientation of interest. The final density parameter utilized in the FE model was the sample set average value of *ρ =* 1271.185 kg/m^3^.

Specific heat (*C_p_*) measurements were performed with a TA Differential Scanning Calorimeter instrument (DSC2500—TA Instruments, New Castle, DE, USA) according to ASTM D3418-21 [54]. Five samples weighing at least 5 mg were tested to verify consistency in the measured response. Table 2 shows a subset of the values utilized in the FE model, which was an average of the five samples from 25 to 225 °C.

Thermal conductivity at room temperature was determined using the transient plane source (TPS) method according to ISO 22007-2 [55] which utilizes direct thermal diffusivity measurement. In total, 81 paired combinations out of 54 samples (18 samples for each X, Y, and Z orientations) were tested at room temperature of approximately 25 °C. Each sample was cut with the dimensions of 17 × 17 × 7 mm^3^. Capturing the temperature dependence of the conductivity response for the same range of temperatures adopted for specific heat characterization is ideal, however, limitations to equipment functionality prevented this level of fidelity. After averaging the measurement data among all samples for each orientation, the orthotropic thermal conductivity values at room temperature adopted in the FE model were 0.59, 0.48. and 0.35 W/m^2^K for the X, Y, and Z directions, respectively.

**Figure 2:**
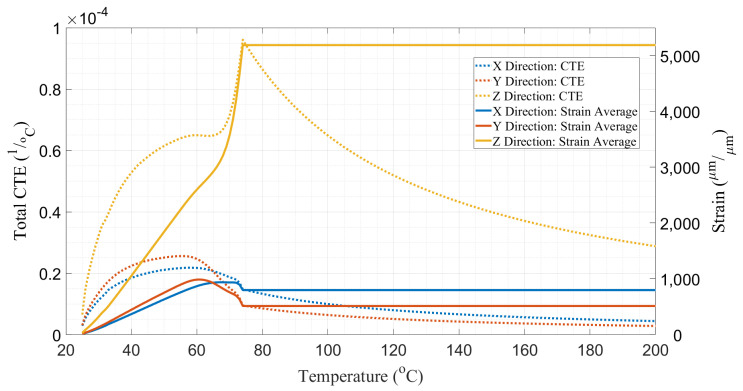
Average of total CTE and strain values out of 5 different PETG/CF samples for x, y, and z orientations.

Coefficient of thermal expansion (CTE) values were obtained by using a TA Thermomechanical Analyzer (TMA Q400—TA Instruments, New Castle, DE, USA) according to ASTM E831-19 [56]. In total, 5 samples for each orientation, X (4.9 × 4.9 × 8.2 mm^3^), Y (4.9 × 4.9 × 6.9 mm^3^), and Z (4.9 × 4.9 × 4.1 mm^3^), were tested. Strain measurements from the test were preserved for temperatures below the glass transition temperature (T_g_ = 74.4 °C) of the material, which was determined according to ASTM D7028-07 [57]. At temperatures above T_g_, thermally-induced strains were assumed to be constant. The CTE for each orientation was obtained by dividing the strain measurement values by the difference between a temperature of interest and the reference temperature of T_ref_ = 20 °C. The average strain curves and their derived CTE curves for each orientation are shown in Figure 2.

Elastic response in X and Z orientations as a function of temperature was measured by a TA Dynamic Mechanical Analyzer (DMA850—TA Instruments, New Castle, DE, USA) according to ASTM D5023-15 [58]. Three rectangular specimens (49 × 2 × 10.6 mm^3^) for X and Z orientations were tested in flexure as a beam. The elastic response in Y orientation was assumed to be the same as the Z orientation response for simplification. Values for shear moduli and Poisson’s ratios were obtained from published tensile and compressive test data [59,60]. The subsequent room temperature orthotropic elastic response was used as a reference definition for the multi-factor approach [61] in order to represent the temperature dependence of the elastic stiffness, as shown in Table 3. The elastic stiffness values were assumed to be constant for temperatures at and above 74.2 °C.

### 2.4. Thermal FE Model of the PETG/CF Wall

Thermal models of the single-bead PETG/CF wall manufactured on the BAAM were implemented in Abaqus/CAE 2021.HF8. The models utilized the Additive Manufacturing (AM) module of Abaqus that drives sequential element activation by means of an event series. An in-house MATLAB code was used to generate the event series from the G-Code-based definition of the toolpath given to the BAAM numerical controller. While the cross-section of the extruded layers is approximately elliptical, for simplicity, the models assume a rectangular bead cross-section.

**Figure 3:**
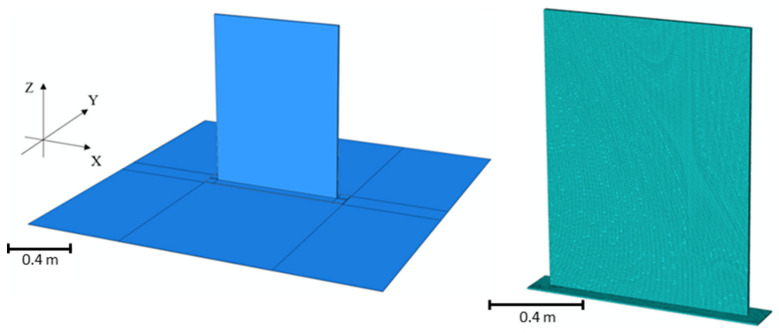
Image of the FE model of the PETG/CF wall on top of the BAAM bed (**left**) and an image of the mesh used on the PETG/CF wall (**right**).

The wall was meshed with linear hexahedral heat transfer elements (DC3D8) with a seed interval set equal to the layer height of 5.076 mm. The same interval was used for element length and width, producing a mesh comprised of cubic elements. Mesh convergence studies were performed separately to ensure the mesh density chosen for this analysis was acceptable. The bed was modeled using the same DC3D8 heat transfer element in direct contact with the brim. The thickness for the bed geometry was 1.6 mm corresponding to the thickness of the ABS sheet placed on the bed for printing. A density of 1140 kg/m^3^, a thermal conductivity of 0.17 W/m^2^K, and a specific heat of 1640 J/kg-K [62] were used for the ABS/CF sheet. Figure 3 shows the FE model including the wall and bed along with an image of the wall mesh.

Thermal analysis used a heat transfer step with a fixed time increment of 10 s. This time-step value was tested separately and selected because it provides a balance between computation time and solution accuracy. The top surface of the bed was assigned a convection coefficient of 2.55 W/m^2^K, which was estimated for a horizontal planar surface [63], and an emissivity value of 0.92 [64]. Ambient temperature for the convective coefficient applied to the ABS/CF bed was captured from thermocouple data at the steady-state regime. A fixed temperature boundary condition of 74.5 °C, as measured by thermocouples installed at the part/bed interface, was set for the bottom and side surfaces of the bed, and the same temperature was used as an initial condition for the entire bed.

The thermal history data from FE models were extracted at selected nodes which correspond to the measured locations of the thermocouples. Synchronization in time was necessary to accurately compare the experimental and model-generated values. Data obtained from thermocouples exhibit a “ramp onset” feature, which is defined as the moment in time when the extrudate is deposited over the thermocouple and a sharp rise in temperature is observed. This feature of the thermocouple time series was aligned with its equivalent nodal activation feature in the FE model. After synchronization, the interpolation of FE data was conducted such that the number of sample points was equal in preparation for root-mean-square (RMS) analysis. Conductance was initially varied in the FE models, and the temperature results were compared to the thermocouple (TC) data obtained at the part/bed interface (C0 and C1). The convection study was then carried out by comparing FE model temperature data with temperature data for all subsequent thermocouples that exhibited good quality contact with the extrudate (C2, C4, C5, C6, C7, AI1, and AI2).

**Figure 4:**
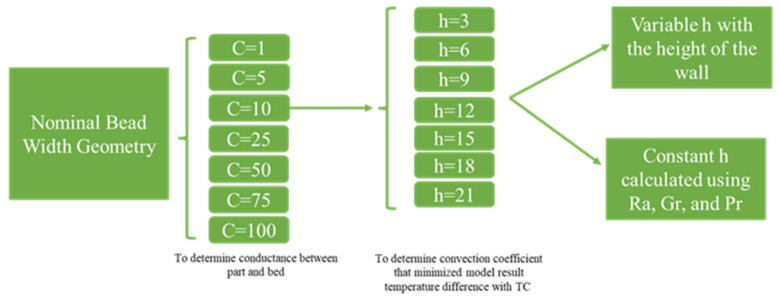
Diagram of the plan followed during conductance and convection study of the FE models.

In the following section, results are presented on how conductance and convection values in the FE model were found from fitting the model with experimental data. Each fitting was assessed based on RMS analysis; final conductance and convection values minimized the error between experimentally obtained thermal histories and their model-predicted equivalents. Several RMS time window sizes from 5 to 300 s were tested, issuing similar results. A visualization of the study progression is shown in Figure 4.

## 3. Results and Discussion

### 3.1. Wall/Bed Thermal Conductance Study

For the conductance study, the convection coefficient applied to the first 10 layers of the wall was assumed to be a constant value of 3 W/m^2^K corresponding to the estimated value for a vertical surface [63]. The ambient temperature definition for the first layer of the wall was set to 74.5 °C to approximate the air temperature near the heated bed, while the ambient temperature for the remaining layers was set to 40 °C, which is approximately the value measured by embedded thermocouples after cooling to steady state. A convection coefficient of −3 W/m^2^K was imposed at the bottom surface of the wall to obtain a “net convection” of zero in that region. Although it is an unphysical value, this approach ensures no convective behavior between the part and bed. This is necessary due to limitations of the Abaqus AM module, which is incapable of natively differentiating between exterior part surfaces where convection occurs and exterior faces of the part that are in contact with the print bed. This negative convective coefficient approach could not be repeated for the radiation boundary condition that is similarly imposed. As a result, the FE model is expected to over-predict cooling at the bottom surface of the part.

**Figure 5:**
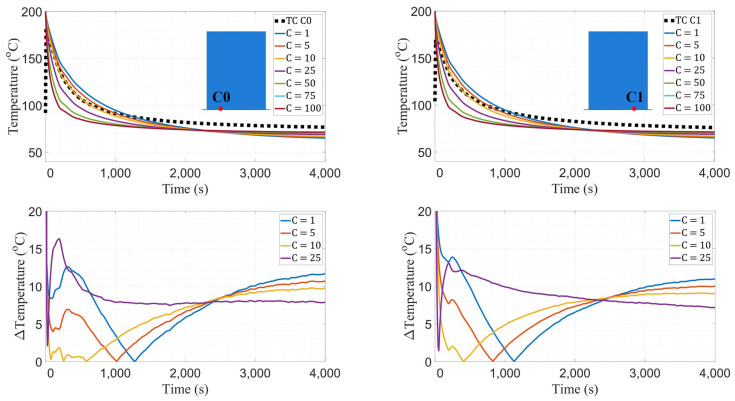
Temperature data comparison (**top**) and RMSE plots (**bottom**) between TCs C0 and C1 and FE model. The plots for C = 75 W/m^2^K and C = 100 W/m^2^K are indistinguishable.

Thermal conductance values of 1, 5, 10, 25, 50, 75, and 100 W/m^2^K were investigated. Temperature data were extracted from two nodes at the interface over a period of 4000 s and compared against data reported by thermocouples C0 and C1. As shown in Figure 5, a conductance (C) value of 10 W/m^2^K produces thermal histories with minimal error. The difference of 5–10 °C at the steady-state regime between experimental and model data is deemed negligible in favor of the early period of cooling because it captures the thermal behavior of layers closer to the bed. For both thermocouples at the interface, temperature curves generated by using C = 10 W/m^2^K exhibited the minimum temperature error until 750 s, corresponding to approximately 5 layers of deposition in the manufacturing process. Figure 5 shows root-mean-square error (RMSE) plots with a five-second window, visualizing that C = 10 W/m^2^K is the conductance value that minimizes error compared to both thermocouples. Accordingly, the convective coefficient study utilizes C = 10 W/m^2^K as the thermal conductance value between the wall and the bed.

### 3.2. Wall Convection Study

One of the most basic problems in the study of heat transfer over external surfaces is the natural convection boundary layer flow over a semi-infinite flat plate [65]. The free convection problem of a non-isothermal vertical plate has been extensively studied by several authors [66,67,68,69]. Analytical equations have been derived to obtain convection coefficients for a vertical plate through Nusselt number equations, which are a function of the Rayleigh and Prandtl numbers [16,70,71]. Although there is a small air inlet near the BAAM bed to help remove vapors, the environment inside the printer is assumed to favor free convection for the purposes of this study.

**Figure 6:**
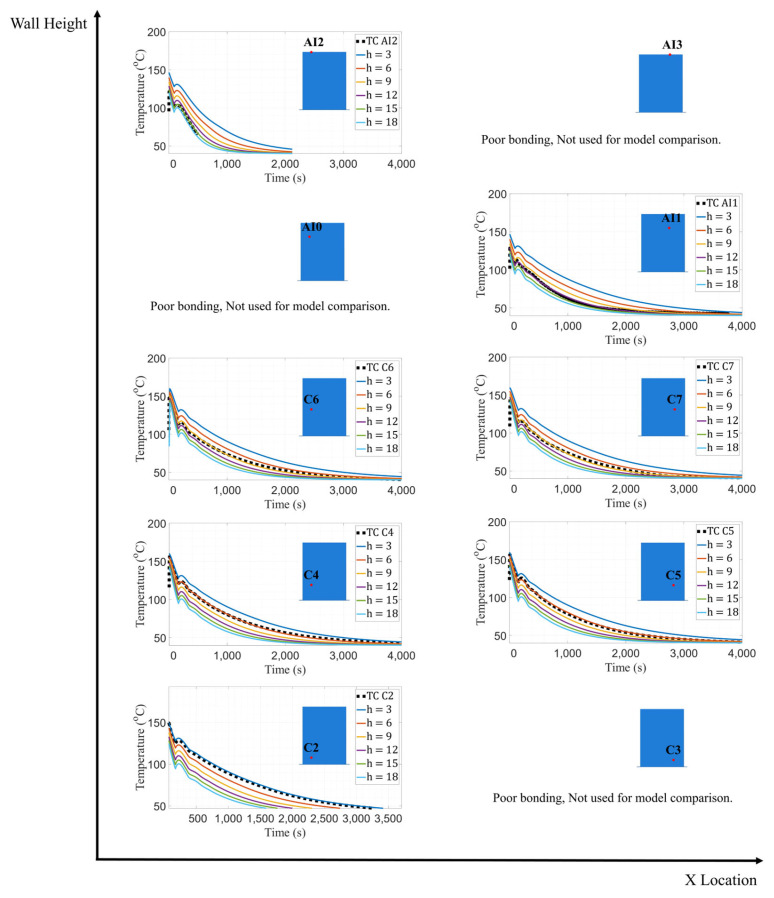
Temperature data comparison between TCs C2, C4, C5, C6, C7, AI1, and AI2 and FE model with varying convection values.

The conductance between the wall and bed was set to 10 W/m^2^K following the results of the previous study. The ambient temperature of the first layer of the wall was maintained at 74.5 °C, while the ambient temperature for the remaining layers was set to 40 °C in similar fashion to the conductance study. A convection coefficient (h) of −3 W/m^2^K was again adopted for the bottom surface of the wall. Several iterations were completed with various convection coefficients of 3, 6, 9, 12, 15, 18, and 21 W/m^2^K for the entire wall to determine the coefficient value which minimized the error between measured and model-predicted temperatures. Data were extracted at the locations of all embedded thermocouples other than C3, AI0 and AI3, which were excluded due to poor bonding. The temperature distribution was extracted at nodes that correspond to the physical location of the thermocouples and was recorded over the duration of the simulation. Model-predicted temperatures for various convection coefficient values are shown in Figure 6.

Table 4 shows that as the vertical position of each thermocouple increases, the convection coefficient that matched experimental data best also tended to increase. These results are corroborated via RMSE analysis. Accordingly, a coefficient value that minimized error at each height was selected to create a distribution of convection coefficients that minimized error over the entire wall. This progression of coefficients as a function of vertical distance from the print bed has also been observed with the use of IR cameras [15].

### 3.3. Wall Residual Stress Study

Temperature and stress distributions predicted by a finite element model assuming a constant convection coefficient were compared against those utilizing a variable convection coefficient to determine the impact on the prediction of residual stresses generated within the part. Model geometry and material properties were identical to those used in the convective coefficient study. For the FE model using constant convection, the coefficient was estimated analytically [70,71] by Equation (1).

(1) $$h=\frac{Nu\times k_{air}}{L}$$

where *k_air_* is the thermal conductivity of the air, L is the wall height, and *Nu* is the average Nusselt number. The average Nusselt number for a vertical isothermal plate is given by Equation (2).

(2) $$Nu={\left\{0.825+\frac{0.387\times {Ra}^{1/6}}{{\left[1+{\left(0.492/Pr\right)}^{9/16}\right]}^{8/27}}\right\}}^{2}$$

where *Ra* is the Rayleigh number (dimensionless number associated with buoyancy-driven flow), *Gr* is the Grashof number (dimensionless number which approximates the ratio of the buoyancy to viscous forces acting on a fluid), and *Pr* is the Prandtl number (ratio of momentum diffusivity to thermal diffusivity), which are each defined by Equation (3), Equation (4), and Equation (5), respectively.

(3) Ra=Gr×Pr

(4) $$Gr=\frac{g\beta \left(T_{film}-T_{\infty }\right)L^{3}}{{\nu_{air}}^{2}}$$

(5) $$Pr=\frac{\nu_{air}}{\alpha_{air}}$$

In Equations (3)–(5), *ν_air_* is the kinematic viscosity of the air, *α_air_* is the thermal diffusivity of the air, *g* is the gravitational constant, β ≈ 1/T_∞_ is the volumetric expansion coefficient of the air, T_film_ is the arithmetic mean between deposition temperature and T_∞_, and T_∞_ is the ambient temperature. The properties of air used in Equations (3)–(5) [72] are given in Table 5. The estimated constant convection coefficient value as calculated with Equation (1) is 11.2 W/m^2^K.

**Figure 7:**
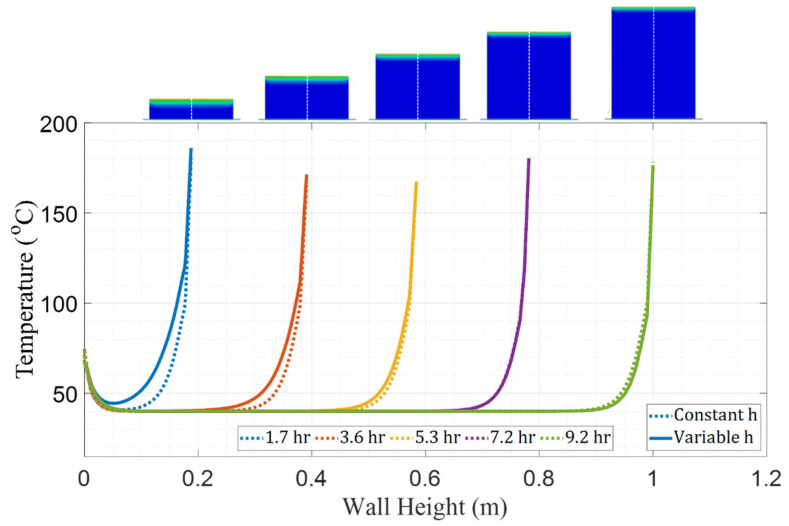
Comparison of temperature distribution across the wall height (dashed white line) at different moments in time between the FE models with constant and variable convection coefficients.

Figure 7 shows a graph of the thermal comparison between FE models with constant and variable convection coefficients. Temperatures were plotted for the nodes that coincide with the dashed white line along the vertical axis of the wall at different moments during the manufacturing process, as shown by images prepended to the top of the graph. Five instances in time were captured corresponding to periods when the wall was at the same height as each respective pair of embedded thermocouples. At Time 1 (1.7 h), the model with constant convection over-predicts cooling by as much as 22 °C, which is consistent with the previous convection study showing that h = 3 W/m^2^K is more suitable than the area-averaged value of h = 11.2 W/m^2^K. At Time 2 (3.6 h), the constant coefficient model also over-predicts cooling, although the difference is reduced to 14 °C at maximum. Although at Time 3, there is still slight overcooling exhibited by the model with constant *h*, temperature curves at Time 3 and Time 4 using the experimentally derived, variable values of *h* closely approximate those based on the constant convection coefficient value. At Time 5, the model with constant *h* begins to under-predict cooling, which is expected because the experimentally derived coefficient value for that height (15 W/m^2^K) is higher than the constant coefficient value, which forces heat dissipation at a faster rate.

**Figure 8:**
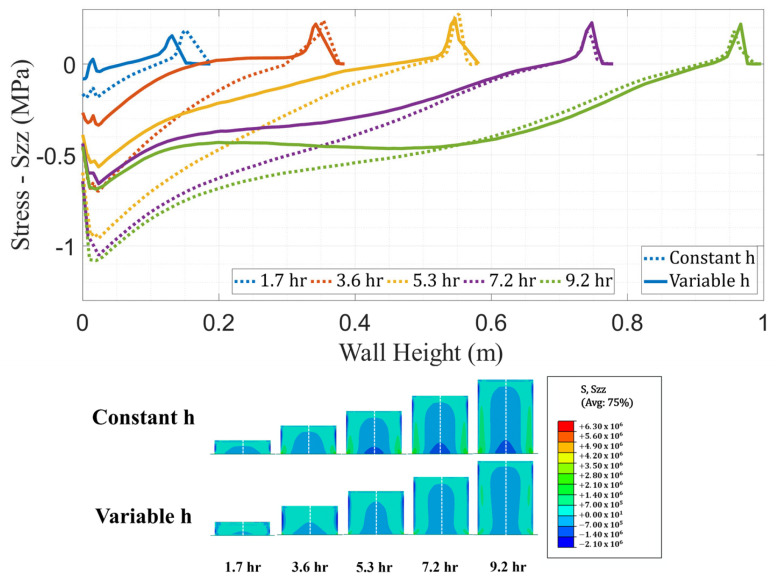
Comparison of stress (Szz) distribution across the wall height (dashed white line) at different moments in time between the FE models with constant and variable convection coefficients.

The results from both thermal models were given as inputs to structural models for residual stress computation and comparison. Structural analyses were executed with an Abaqus AM module with the CTE and elastic stiffness constants determined through material characterization. The models utilized a 100 s time step and 8-node linear hexahedral elements with reduced integration with hourglass control (C3D8R). Mesh connectivity and nodal positions were retained from the thermal model. Fixed boundary conditions were applied at the base of the wall to simulate a rigid adhesion to the bed. Stresses were measured from the integration points in elements along the height of the wall at the same instances in time that temperature data were extracted. Figure 8 shows the comparison of stresses in the vertical direction (Szz) between the models with constant and variable convection coefficients. This stress component was selected because it represents the normal inter-layer stress between beads, which is a critical parameter when assessing the potential for inter-layer de-bonding during manufacturing or after a part is completed.

A direct comparison between strains calculated by the FE model and experimentally obtained strains is not possible due to the lack of available data. As such, the following residual stress predictions are not experimentally validated. However, the simulation-based comparison shows that the model with constant *h* predicts higher magnitudes of Szz when compared to the variable *h* model with maximum discrepancies in the lower regions of the wall. In Figure 8, the Szz stress distributions show higher stress concentration at the base of the wall for the model with a constant convection coefficient. 

The PETG/CF tensile strength was measured in accordance with ASTM D638 [73] with an average value for Szz of 9 MPa. Peak stress values from both constant and variable *h* models were below this threshold, which is consistent with the observation that no crack formation or de-bonding occurred in the wall. Nevertheless, the comparatively higher stresses presented by the constant *h* model could impact predictions of crack formation in a part. In summary, thermal history and stress analysis results show that neglecting the variation of *h* could impose inaccuracies on the quality of predictions generated by the FE model.

### 3.4. Extension to Other Vertical Walls Printed on BAAM

In the convective study presented previously, the convection coefficients for the PETG/CF wall were found by means of comparing model and experimental temperature data along the height of the wall. The same study was conducted for an ABS/CF wall manufactured with the BAAM consisting of the same geometry. Different manufacturing parameters, such as layer time, were selected and accounted for in the FE model through an updated event series input. Thermocouple placement in the ABS/CF wall was executed in similar fashion to the PETC/CF equivalent. The objective for repeating the study in a different material system was to discover whether the ABS/CF case analysis would result in a similar *h* trend found for the PETG/CF wall, thus adding reliability to those results. Repetition of the study produced similar convection coefficient variations as presented in Table 6. Although the trend found for the ABS/CF wall study is not linear, the *h* values are close in magnitude to those reported in Table 4 even when taking into consideration the differences in material systems and printing process conditions.

The means of providing a generalized solution that could potentially be applied to any vertical wall printed on the BAAM is now presented. Researchers commonly report generalized solutions through correlation equations, which are expressions developed to provide approximations to local convection coefficients for specific conditions such as vertical and horizontal plates, enclosures, etc. These equations are obtained through numerical, analytical, and experimental measurement [70] methods. The assumption in using correlation equations for natural convection in process simulations is that natural convection occurs instantaneously during the manufacturing process [44]. Correlation equations are generally posed in terms of dimensionless numbers such as the Rayleigh and Prandtl numbers given by Equations (3) and (5).

Analytical solutions obtained for the laminar boundary layer problem of both isothermal and non-isothermal vertical plates provide expressions for the local Nusselt number (*Nu_z_*) in the same general format as Equation (6) [44,74]. In this study, the Nusselt number was found by applying the relationship between (*Nu_z_*) and *h* given by Equation (1), while the Rayleigh number was found by using the surface temperature data as a function of height from the FE model with the progression of convection coefficients that minimized error in relation to experimental data from the PETG/CF wall. In Equation (4), before applying Equation (3), the Prandtl number was simply calculated by Equation (5) given the air properties according to the surface temperature data from the variable convection coefficient wall model.

(6) $${Nu}_{z}=A\cdot {Ra}_{z}^{m}\cdot {Pr}_{z}^{n}$$

**Figure 9:**
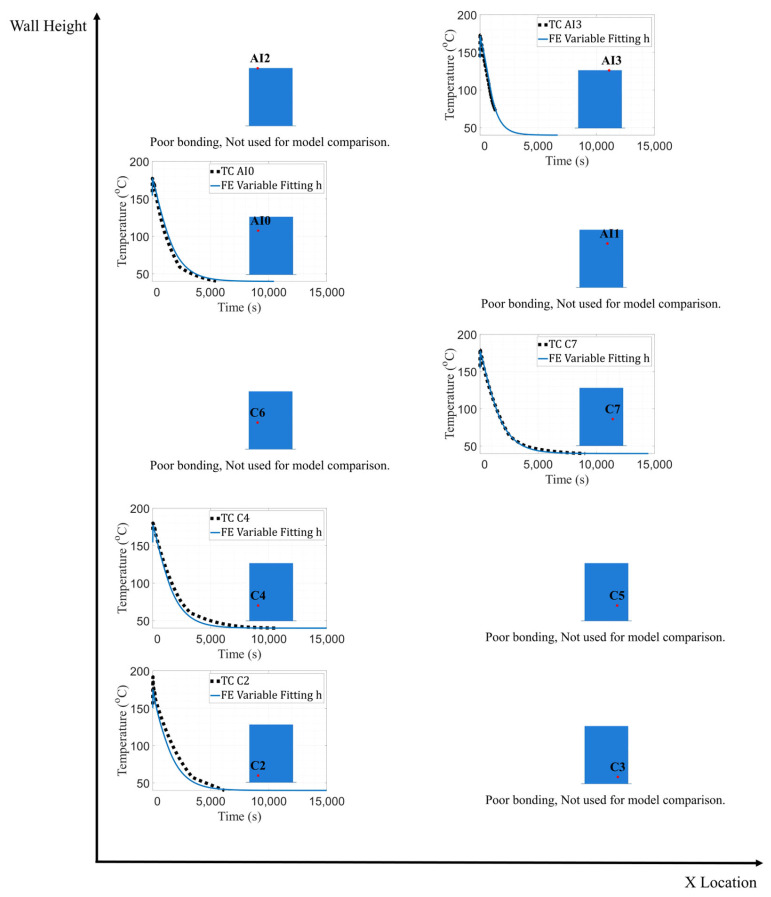
Temperature data comparison between TCs C2, C3, C4, C5, C6, C7, AI0, AI1, AI2, and AI3 and FE model using the variable convection coefficients found by Equation (6).

The unknown parameters A (521.22), m (0.26), and n (5.00) were found by utilizing the nonlinear least-squares curve-fitting method in MATLAB. These parameters were then used to derive convection coefficient values which were applied along the height of the ABS/CF wall via Equation (1). This new distribution of *h* was then used in the FE model of the ABS/CF wall, and the temperature results were compared with the equivalent experimentally obtained thermocouple data. The comparison between thermocouple and FE data shown in Figure 9 displays good agreement as corroborated by RMS analysis. The result of this analysis demonstrates that the correlation equation derived from PETG/CF data can potentially be applied to other vertical walls printed on the BAAM, thus extending the solution found to situations having different material systems and process conditions.

## 4. Conclusions

This work successfully demonstrates how to obtain in situ temperature data from a additively manufactured PETG/CF vertical wall using thermocouples embedded between layers in the part. Combined with comprehensive material characterization, thermal history data were given as inputs for the development of a predictive FE process model in Abaqus CAE. The following can be concluded:

1. There were significant differences in temperature predictions between FE models that applied constant or variable convection coefficients. During the early stages of manufacturing, the model with a constant convection coefficient over-predicts cooling and gradually transitions to an under-prediction of cooling rate.
2. The progression of convection coefficients which minimized error in comparison with experimental data increased with the height of the wall. This may be due to the difference in air flow at regions of the wall at increasing heights above the BAAM print bed. The linearity of the increase of convection coefficient is a compelling result. However, this linear behavior cannot be extended as a rule, which is demonstrated by independent predictions of the thermal history from the ABS/CF wall manufactured with similar process conditions.
3. Significant differences were found in stress predictions between both models. The model that used a constant convection coefficient predicted normal stresses in the vertical direction at locations closest to the bed and during all stages of manufacturing that were nearly double those predicted with the variable convection coefficient model. Although cracks were not observed in the part and both models predicted stresses below the material strength, these results suggest that accurate thermal modeling is crucial for the prediction of residual stresses and, consequently, part integrity during and immediately after the manufacturing process.
4. Regarding material characterization, adding thermal conductivity data as a function of temperature to the FE thermal model may further improve model predictions.
5. The analysis of data generated by the PETG/CF wall experiment enabled the derivation of a correlation equation between convection coefficient and air properties that can be applied to other vertical surfaces manufactured with the BAAM.

## Figures and Tables

**Table 1:** PETG/CF wall dimensions and printing process information.

Dimensions	Value	Printing Process Information	Value
Wall Height (m)	1	Deposition Temperature (°C)	200
Wall Length (m)	0.75	Bed Set Temperature (°C)	90
Bead Width (mm)	15.875	Dryer Temperature (°C)	60
Bead Height (mm)	5.08	Feed Rate (m/min)	3.24
Bead Count	1	Deposition/Layer Time (s)	<168/13.9
Layer Count	197	Manufacturing Time (hours)	<10

**Table 2:** Average of specific heat out of five different PETG/CF samples for x, y, and z orientations.

Temperature (°C)	25	60	75	90	125	200	225
C_p_ (J/kg°C)	763.8	903.2	1154.4	1181.6	1268.8	1442.4	1496.6

**Table 3:** Orthotropic elastic properties as a function of temperature used in the FE model.

Temperature (°C)	E_xx_ (MPa)	E_yy_ (MPa)	E_zz_ (MPa)	G_xy_ (MPa)	G_xz_ (MPa)	G_yz_ (MPa)	ν_xy_	ν_yz_	ν_xz_
20	12,100 ± 15	2720	2720 ± 10	1110	1110	1134	0.38	0.39	0.38
32.8	6876 ± 18	2612	2612 ± 12	2509	2509	972	0.32	0.34	0.32
44.3	6761 ± 19	2569	2569 ± 12	2467	2467	955	0.32	0.34	0.32
54.2	6640 ± 24	2523	2523 ± 13	2423	2423	938	0.32	0.34	0.32
64.5	6389 ± 25	2427	2427 ± 13	2332	2332	903	0.32	0.34	0.32
74.2	4289 ± 25	1630	1630 ± 13	1565	1565	606	0.32	0.34	0.32

**Table 4:** Best fitted value of convection coefficient (h) at each embedded thermocouple height for the FE model.

Wall Height (m)	Convection Coefficient—h (W/m^2^K)
0.19	3
0.39	6
0.58	9
0.78	12
0.98	15

**Table 5:** Properties used in the calculation of the constant convection coefficient.

Properties	Value
Ambient Temperature (°C)	40
Deposition Temperature (°C)	200
Wall Height (m)	1
Air Density (kg/m^3^)	1.127
Air Thermal Diffusivity (m^2^/s)	2.346 × 10^−5^
Air Kinetic Viscosity (m^2^/s)	1.702 × 10^−5^
Air Thermal Conductivity (W/mK)	0.02662
Gravitational Constant (m/s^2^)	9.81

**Table 6:** Best fitted value of convection coefficient (*h*) for each inter-layer TC height (*h* versus wall height) for the FE model of ABS/CF.

Wall Height (m)	Convection Coefficient—h (W/m^2^K)
0.19	3
0.39	3
0.58	6
0.78	9
0.98	15

## Data Availability

Not applicable.

## References

[1] Post B.K., Chesser P.C., Lind R.F., Roschli A., Love L.J., Gaul K.T., Sallas M., Blue F., Wu S. (2019). Using Big Area Additive Manufacturing to Directly Manufacture a Boat Hull Mould. Virtual Phys. Prototyp..

[2] Hassen A.A., Springfield R., Lindahl J., Post B., Love L., Duty C., Vaidya U., Pipes R.B., Kunc V. The Durability of Large-Scale Additive Manufacturing Composite Molds. Proceedings of the CAMX Conference Proceedings.

[3] Al Jassmi H., Al Najjar F., Mourad A.-H.I. (2018). Large-Scale 3D Printing: The Way Forward. IOP Conf. Ser. Mater. Sci. Eng..

[4] Holshouser C., Newell C., Palas S., Love L.J., Kunc V., Lind R.F., Lloyd P.D., Rowe J.C., Blue C.A., Duty C.E. (2013). Out of Bounds Additive Manufacturing. Adv. Mater. Process..

[5] Love L.J., Kunc V., Rios O., Duty C.E., Elliott A.M., Post B.K., Smith R.J., Blue C.A. (2014). The Importance of Carbon Fiber to Polymer Additive Manufacturing. J. Mater. Res..

[6] Love L.J. (2015). Utility of Big Area Additive Manufacturing (BAAM) for the Rapid Manufacture of Customized Electric Vehicles.

[7] Duty C.E., Kunc V., Compton B., Post B., Erdman D., Smith R., Lind R., Lloyd P., Love L. (2017). Structure and Mechanical Behavior of Big Area Additive Manufacturing (BAAM) Materials. Rapid Prototyp. J..

[8] Ferrini-Mundy J., Varahramyan K. (2023). 2022 Research Report: R1 Global Impact—Local Relevance.

[9] Lee J.-Y., An J., Chua C.K. (2017). Fundamentals and Applications of 3D Printing for Novel Materials. Appl. Mater. Today.

[10] Shofner M.L., Lozano K., Rodríguez-Macías F.J., Barrera E.V. (2003). Nanofiber-reinforced Polymers Prepared by Fused Deposition Modeling. J. Appl. Polym. Sci..

[11] Ahn S., Montero M., Odell D., Roundy S., Wright P.K. (2002). Anisotropic Material Properties of Fused Deposition Modeling ABS. Rapid Prototyp. J..

[12] Hoskins D., Kunc V., Hassen A., Lindahl J., Duty C. Characterizing Thermal Expansion of Large-Scale 3D Printed Parts. Proceedings of the SAMPE 2019.

[13] Advani S.G., Tucker C.L. (1987). The Use of Tensors to Describe and Predict Fiber Orientation in Short Fiber Composites. J. Rheol..

[14] Compton B.G., Post B.K., Duty C.E., Love L., Kunc V. (2017). Thermal Analysis of Additive Manufacturing of Large-Scale Thermoplastic Polymer Composites. Addit. Manuf..

[15] Brenken B., Barocio E., Favaloro A., Kunc V., Pipes R.B. (2019). Development and Validation of Extrusion Deposition Additive Manufacturing Process Simulations. Addit. Manuf..

[16] Brenken B. (2017). Extrusion Deposition Additive Manufacturing of Fiber Reinforced Semi-Crystalline Polymers.

[17] Zhang Y., Chou Y.K. (2006). Three-Dimensional Finite Element Analysis Simulations of the Fused Deposition Modelling Process. Proc. Inst. Mech. Eng. Part B J. Eng. Manuf..

[18] Lieneke T., Denzer V., Adam G.A.O., Zimmer D. (2016). Dimensional Tolerances for Additive Manufacturing: Experimental Investigation for Fused Deposition Modeling. Procedia CIRP.

[19] Alaimo G., Marconi S., Costato L., Auricchio F. (2017). Influence of Meso-Structure and Chemical Composition on FDM 3D-Printed Parts. Compos. Part B Eng..

[20] Ziemian C., Sharma M., Ziemian S. (2012). Anisotropic Mechanical Properties of ABS Parts Fabricated by Fused Deposition Modelling. Mech. Eng..

[21] Soleyman E., Aberoumand M., Soltanmohammadi K., Rahmatabadi D., Ghasemi I., Baniassadi M., Abrinia K., Baghani M. (2022). 4D Printing of PET-G via FDM Including Tailormade Excess Third Shape. Manuf. Lett..

[22] Soleyman E., Rahmatabadi D., Soltanmohammadi K., Aberoumand M., Ghasemi I., Abrinia K., Baniassadi M., Wang K., Baghani M. (2022). Shape Memory Performance of PETG 4D Printed Parts under Compression in Cold, Warm, and Hot Programming. Smart Mater. Struct..

[23] Dinwiddie R.B., Love L.J., Rowe J.C., Stockton G.R., Colbert F.P. (2013). Real-Time Process Monitoring and Temperature Mapping of a 3D Polymer Printing. Proceedings of the SPIE 8705, Thermosense: Thermal Infrared Applications XXXV, Baltimore, MD, USA, 22 May 2013.

[24] Dinwiddie R.B., Kunc V., Lindal J.M., Post B., Smith R.J., Love L., Duty C.E., Colbert F.P., Hsieh S.-J. (2014). Infrared Imaging of the Polymer 3D-Printing Process. Proceedings of the SPIE 9105, Thermosense: Thermal Infrared Applications XXXVI, Baltimore, MD, USA, 12 June 2014.

[25] Meraz Trejo E., Jimenez X., Billah K.M.M., Seppala J., Wicker R., Espalin D. (2020). Compressive Deformation Analysis of Large Area Pellet-Fed Material Extrusion 3D Printed Parts in Relation to in Situ Thermal Imaging. Addit. Manuf..

[26] Planinsic G. (2011). Infrared Thermal Imaging: Fundamentals, Research and Applications. Eur. J. Phys..

[27] Kousiatza C., Tzetzis D., Karalekas D. (2019). In-Situ Characterization of 3D Printed Continuous Fiber Reinforced Composites: A Methodological Study Using Fiber Bragg Grating Sensors. Compos. Sci. Technol..

[28] Kousiatza C., Chatzidai N., Karalekas D. (2017). Temperature Mapping of 3D Printed Polymer Plates: Experimental and Numerical Study. Sensors.

[29] Chin R.K., Beuth J.L., Amon C.H. (1996). Thermomechanical Modeling of Molten Metal Droplet Solidification Applied to Layered Manufacturing. Mech. Mater..

[30] Talagani M., DorMohammadi S., Dutton R., Godines C., Kumar Baid H., Abdi F., Kunc V., Compton B.G., Simunovic S., Duty C. (2015). Numerical Simulation of Big Area Additive Manufacturing (3D Printing) of a Full Size Car. SAMPE J..

[31] Bhandari S., Lopez-Anido R. (2018). Finite Element Analysis of Thermoplastic Polymer Extrusion 3D Printed Material for Mechanical Property Prediction. Addit. Manuf..

[32] Bhandari S., Lopez-Anido R.A. (2020). Discrete-Event Simulation Thermal Model for Extrusion-Based Additive Manufacturing of PLA and ABS. Materials.

[33] Zhang J., Wang X.Z., Yu W.W., Deng Y.H. (2017). Numerical Investigation of the Influence of Process Conditions on the Temperature Variation in Fused Deposition Modeling. Mater. Des..

[34] Stockman T., Schneider J.A., Walker B., Carpenter J.S. (2019). A 3D Finite Difference Thermal Model Tailored for Additive Manufacturing. JOM.

[35] Bhandari S., Lopez-Anido R.A. (2023). Coupled Thermo-Mechanical Numerical Model to Minimize Risk in Large-Format Additive Manufacturing of Thermoplastic Composite Designs. Prog. Addit. Manuf..

[36] Zhou Y., Nyberg T., Xiong G., Liu D. Temperature Analysis in the Fused Deposition Modeling Process. Proceedings of the 2016 3rd International Conference on Information Science and Control Engineering (ICISCE).

[37] Ji L.B., Zhou T.R. (2010). Finite Element Simulation of Temperature Field in Fused Deposition Modeling. Adv. Mater. Res..

[38] D’Amico A., Peterson A.M. (2018). An Adaptable FEA Simulation of Material Extrusion Additive Manufacturing Heat Transfer in 3D. Addit. Manuf..

[39] Bhandari S., Lopez-Anido R. (2019). Finite Element Modeling of 3D-Printed Part with Cellular Internal Structure Using Homogenized Properties. Prog. Addit. Manuf..

[40] Deering R.A. (2018). Additive Manufacturing Part Level Distortion Sensitivity Analysis within Abaqus on a Thin Walled, Tubular Structure. https://www.3ds.com/fileadmin/PRODUCTS-SERVICES/SIMULIA/Resources-center/PDF/2018-SaoE-Additive_Manufacturing_Part_Level_Distortion_Sensitivity_Analysis_within_Abaqus_on_a_Thin-walled__Tubular_Structure.pdf.

[41] Cattenone A., Morganti S., Alaimo G., Auricchio F. (2019). Finite Element Analysis of Additive Manufacturing Based on Fused Deposition Modeling: Distortions Prediction and Comparison with Experimental Data. J. Manuf. Sci. Eng..

[42] Courter B., Savane V., Bi J., Dev S., Hansen C.J. Finite Element Simulation of the Fused Deposition Modelling Process. Proceedings of the NAFEMS World Congress.

[43] Favaloro A.J., Brenken B., Barocio E., Pipes R.B. Simulation of Polymeric Composites Additive Manufacturing Using Abaqus. Proceedings of the Science in the Age of Experience.

[44] Vaca E.B. (2018). Fusion Bonding of Fiber Reinforced Semi-Crystalline Polymers in Extrusion Deposition Additive Manufacturing.

[45] Nycz A., Lee Y., Noakes M., Ankit D., Masuo C., Simunovic S., Bunn J., Love L., Oancea V., Payzant A. (2021). Effective Residual Stress Prediction Validated with Neutron Diffraction Method for Metal Large-Scale Additive Manufacturing. Mater. Des..

[46] Guessasma S., Belhabib S., Nouri H. (2019). Printability and Tensile Performance of 3D Printed Polyethylene Terephthalate Glycol Using Fused Deposition Modelling. Polymers.

[47] Dolzyk G., Jung S. (2019). Tensile and Fatigue Analysis of 3D-Printed Polyethylene Terephthalate Glycol. J. Fail. Anal. Prev..

[48] Billah K.M.M., Lorenzana F.A.R., Martinez N.L., Wicker R.B., Espalin D. (2020). Thermomechanical Characterization of Short Carbon Fiber and Short Glass Fiber-Reinforced ABS Used in Large Format Additive Manufacturing. Addit. Manuf..

[49] Rios O., Carter W., Post B., Lloyd P., Fenn D., Kutchko C., Rock R., Olson K., Compton B. (2018). 3D Printing via Ambient Reactive Extrusion. Mater. Today Commun..

[50] Huang H., Ma N., Chen J., Feng Z., Murakawa H. (2020). Toward Large-Scale Simulation of Residual Stress and Distortion in Wire and Arc Additive Manufacturing. Addit. Manuf..

[51] Nycz A., Kishore V., Lindahl J., Duty C., Carnal C., Kunc V. (2020). Controlling Substrate Temperature with Infrared Heating to Improve Mechanical Properties of Large-Scale Printed Parts. Addit. Manuf..

[52] Colón Quintana J.L., Slattery L., Pinkham J., Keaton J., Lopez-Anido R.A., Sharp K. (2022). Effects of Fiber Orientation on the Coefficient of Thermal Expansion of Fiber-Filled Polymer Systems in Large Format Polymer Extrusion-Based Additive Manufacturing. Materials.

[53] (2020). Standard Test Methods for Density and Specific Gravity (Relative Density) of Plastics by Displacement.

[54] (2021). Standard Test Method for Transition Temperatures and Enthalpies of Fusion and Crystallization of Polymers by Differential Scanning Calorimetry.

[55] (2022). Plastics—Determination of Thermal Conductivity and Thermal Diffusivity—Part 2: Transient Plane Source Method.

[56] (2019). Standard Test Method for Linear Thermal Expansion of Solid Materials by Thermomechanical Analysis.

[57] (2015). Standard Test Method for Glass Transition Temperature (DMA Tg) of Polymer Matrix Composites by Dy-Namic Mechanical Analysis (DMA).

[58] (2016). Standard Test Method for Plastics: Dynamic Mechanical Properties: In Flexure (Three-Point Bending).

[59] Steva B., Warren K., Seigars C., Helten B. Evaluation of the Shear Behavior of CF-PETG and CF-PC Coupons Manufactured Using Large-Scale Additive Manufacturing Processes. Proceedings of the ECCM20—The 20th European Conference on Composite Materials.

[60] Seigars C., Warren K., Steva B., Murphy C., Helten B. Characterizing the Tensile and Compressive Behavior of PETG/CF and PC/CF Manufactured Using Large Scale Additive Processes. Proceedings of the ECCM20—The 20th European Conference on Composite Materials.

[61] Kim P., Baid H., Hassen A., Kumar A., Lindahl J., Hoskins D., Ajinjeru C., Duty C., Yeole P., Vaidya U. Analysis on Part Distortion and Residual Stress in Big Area Additive Manufacturing with Carbon Fiber-Reinforced Thermoplastic Using Dehomogenization Technique. Proceedings of the Composites and Advanced Materials Expo (CAMX 2019).

[62] Choo K., Friedrich B., Daugherty T., Schmidt A., Patterson C., Abraham M.A., Conner B., Rogers K., Cortes P., MacDonald E. (2019). Heat Retention Modeling of Large Area Additive Manufacturing. Addit. Manuf..

[63] Morgan R.V., Stowers Reid R., Baker A.M., Lucero B., Bernardin J.D. (2017). Emissivity Measurements of Additively Manufactured Materials.

[64] Na T.Y. (1977). Numerical Solution of Natural Convection Flow Past a Non-Isothermal Vertical Flat Plate. Appl. Sci. Res..

[65] Chen T.Y.W., Wollersheim D.E. (1973). Free Convection at a Vertical Plate with Uniform Flux Condition in Non-Newtonian Power-Law Fluids. J. Heat Transf..

[66] Sparrow E.M., Gregg J.L. (1956). Laminar Free Convection from a Vertical Plate with Uniform Surface Heat Flux. J. Fluids Eng..

[67] Touloukian Y.S., Hawkins G.A., Jakob M. (1948). Heat Transfer by Free Convection from Heated Vertical Surfaces to Liquids. J. Fluids Eng..

[68] Cheesewright R. (1968). Turbulent Natural Convection from a Vertical Plane Surface. J. Heat Transf..

[69] Churchill S.W., Chu H.H.S. (1975). Correlating Equations for Laminar and Turbulent Free Convection from a Vertical Plate. Int. J. Heat Mass Transf..

[70] Incropera F.P., DeWitt D.P., Bergman T.L., Lavine A.S. (2007). Fundamentals of Heat and Mass Transfer, 6th ed.

[71] Çengel Y.A., Ghajar A.J. (2020). Heat and Mass Transfer: Fundamentals and Applications.

[72] Properties of Air at 1 Atm Pressure. Engineers Edge. https://www.engineersedge.com/physics/properties_of_air_at_1_atm_pressure_13828.htm.

[73] (2022). Standard Test Method for Tensile Properties of Plastics.

[74] Havet M., Blay D. (1999). Natural Convection over a Non-Isothermal Vertical Plate. Int. J. Heat Mass Transf..

